# Consumption of resistant starch decreases postprandial lipogenesis in white adipose tissue of the rat

**DOI:** 10.1186/1475-2891-5-25

**Published:** 2006-09-20

**Authors:** Janine A Higgins, Marc A Brown, Leonard H Storlien

**Affiliations:** 1 University of Colorado Health Sciences Center, Denver, Colorado 80262, USA; 2 Metabolic Research Centre, Faculty of Health & Behavioural Sciences, University of Wollongong, NSW 2522, Australia; 3 AstraZeneca International, Mölndal, Sweden

## Abstract

Chronic consumption of diets high in resistant starch (RS) leads to reduced fat cell size compared to diets high in digestible starch (DS) in rats and increases total and meal fat oxidation in humans. The aim of the present study was to examine the rate of lipogenesis in key lipogenic organs following a high RS or DS meal. Following an overnight fast, male Wistar rats ingested a meal with an RS content of 2% or 30% of total carbohydrate and were then administered an i.p bolus of 50 μCi ^3^H_2_O either immediately or 1 hour post-meal. One hour following tracer administration, rats were sacrificed, a blood sample collected, and the liver, white adipose tissue (WAT), and gastrocnemius muscle excised and frozen until assayed for total ^3^H-lipid and ^3^H-glycogen content. Plasma triglyceride and NEFA concentrations and ^3^H-glycogen content did not differ between groups. In all tissues, except the liver, there was a trend for the rate of lipogenesis to be higher in the DS group than the RS group which reached significance only in WAT at 1 h (p < 0.01). On a whole body level, this attenuation of fat deposition in WAT in response to a RS diet could be significant for the prevention of weight gain in the long-term.

## Findings

It has been reported that chronic resistant starch (RS) feeding in rats causes a decrease in adipocyte cell size, a decrease in fatty acid synthase expression, and reduced whole-body weight gain relative to digestible starch (DS) feeding [1,2]. Additionally, in healthy adults, a single RS meal caused a substantial elevation in total and meal fat oxidation compared to a DS meal [3]. These data suggest that RS intake may influence postprandial lipid metabolism. The aim of the present study was to examine the rate of lipogenesis in key lipogenic organs acutely following a RS or DS meal.

Male Wistar rats (*Rattus norvegicus*) were obtained from the Animal Resource Center (Murdoch, Western Australia) and were housed in groups of three at the University of Wollongong, Animal House. The rats were maintained at 22°C on a 12-h light/dark cycle (light cycle from 0700–1900 h), with free access to a standard laboratory chow (Young Stock Feed, Young, Australia) and water. The study was conducted according to the National Health and Medical Research Council (NH&MRC; Australia) code of practice for the care and use of animals for scientific purposes. Test Diets were prepared as previously described [5]. As a percentage of total energy, all diets contained 67% carbohydrate (57% starch; 10% sucrose), 22% protein, and 11% fat. All diets were identical in composition except for the percentage of RS and DS included in the starch component. For the RS diet, the starch used was a natural high amylose starch, Hi-Maize 957™ (National Starch and Chemical Co), which is 60% amylose/40% amylopectin, versus waxy cornstarch, which is 0% amylose/100% amylopectin, for the DS diet. Diets were presented to the animals in an unprocessed, unpelleted form so the starches were not subjected to cooking or extrusion. The dietary fiber level of the starches was determined using the Association of Analytical Chemistry (AOAC) enzymatic-gravimetric method. Total dietary fiber (dry solids) was lower (approximately 1%) for the DS starch than the RS starch which contained 19.3% fiber.

Two weeks prior to a meal test, rats were presented with test diet ad libatum overnight to familiarize them with this diet and prevent neophobia on the test day. Twice over the next week, rats were starved for 5 h then placed in testing cages and presented with a meal of similar size to that which they would receive during the meal test (approximately 0.4 g of diet). Rats had no exposure to test diet or disruption of normal eating habits for seven days prior to the meal test. For all tests, rats were starved overnight (12–15 hours). The following morning, rats were placed in 25 × 25-cm wire testing cages and allowed 30 min to acclimate before testing commenced. The rat was then presented with a test meal of 1.0 g carbohydrate/kg body weight (1.49 g diet/kg body weight). Rats were allowed 15 minutes to consume all food presented to them. Rats were injected with 50 μCi ^3^H_2_O either immediately following food consumption (1 h groups) or 1 h post-ingestion (2 h groups). One hour after ^3^H_2_O administration, rats were sacrificed by i.p. injection of 250 mg/kg pentobarbitone sodium. 400 μl blood was collected and the gastrocnemius muscle, liver, epidydimal white adipose tissue (WAT), and interscapular brown adipose tissue (BAT) were excised and immediately freeze-clamped. All tissues were stored at -80°C until assayed for lipid and glycogen content. Plasma was pipetted into fresh tubes and stored at -20°C for subsequent analysis of triglyceride and non-esterified fatty acid (NEFA) concentrations.

All plasma samples were assayed in duplicate. Plasma triglycerides were measured using a colorimetric assay kit supplied by Boehringer Mannheim (Germany), according to the manufacturer's instructions. Plasma NEFAs were estimated using a NEFA C kit (Wako Pure Chemicals Inc., Japan). All colorimetric assays were analyzed using a BioRad (model 550) microplate reader (Hercules, CA).

Estimation of total lipogenesis and glycogenesis was conducted as previously described [4]. Briefly, 100–200 mg of tissue was weighed out and completely digested in 1 M KOH by heating at 70°C for 40 min. Half of this tissue digest was spotted onto filter paper, air dried, and glycogen precipitated using ethanol. The remainder of the tissue digest was added to a chloroform/methanol mixture, agitated and the lipid extracted using three hexane washes. Pooled hexane layers from each tissue were dried under nitrogen, suspended in scintillant and counted. Note that glycogen measurements were not performed on adipose tissue samples due to their very low total glycogen content.

Results are expressed as means ± SEM using Statistics for Windows (Analytical Software, Tallahassee, FL), unless otherwise indicated. Statistical differences between diet groups were determined using an ANOVA with repeated measures and protected least significant difference (PLSD) test for comparison of means.

There was no difference in body weight between the groups used (330 ± 18 g and 328 ± 20 g for the DS and RS groups, reapectively). All animals ate at least 87% of the diet presented to them. There was no difference in total test meal consumption between groups. Animals consumed 93% (0.376 g), 95% (0.384 g), 92% (0.372 g), and 96% (0.388 g) of food presented for the RS 1 h, DS 1 h, RS 2 h, DS 2 h groups, respectively.

**Figure 1:**
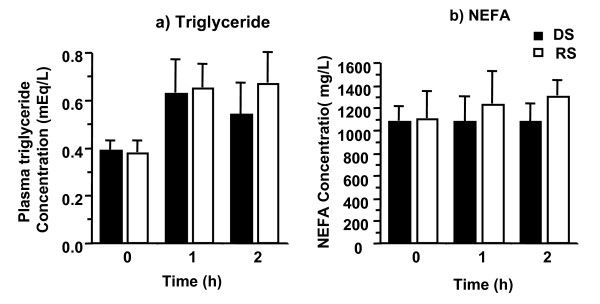
Postprandial plasma triglyceride (a) and non-esterified fatty acid (NEFA; b) concentrations. n = 8 for all but the RS 1 h group in which n = 7.

Plasma triglyceride and NEFA concentrations did not differ between groups (Figure 1). Triglyceride concentrations at baseline, 1 h, and 2 h were 0.39 ± 0.04, 0.63 ± 0.11, and 0.54 ± 0.11 mmol/L for the DS group vs 0.38 ± 0.05, 0.65 ± 0.07, and 0.67 ± 0.09 mmol/L for the RS group. NEFA data showed similar trends.

**Figure 2:**
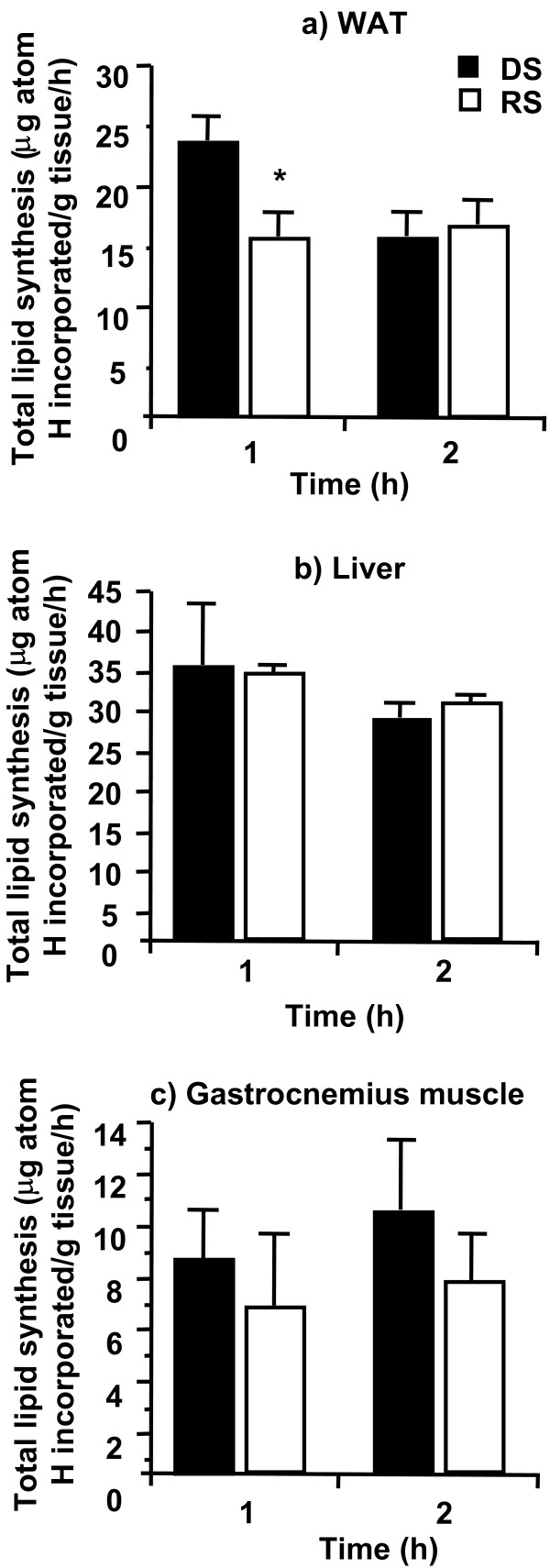
Rate of lipogenesis in a) white adipose tissue (WAT), b) liver, and c) gastrocnemius muscle. n = 8 for all but the RS 1 h group in which n = 7. * p = 0.009 for a difference from the DS group at the same time point.

In all tissues, except the liver, there was a trend for the rate of lipogenesis to be higher in the DS group than the RS group. This trend reached significance only in WAT at 1 h (p < 0.01; Figure 2). There was no significant difference in the rate of glycogensis in the liver or gastrocnemius in response to a DS or RS meal at any time point investigated (data not shown).

The amount of carbohydrate fed per meal in this study is comparable to that used in oral glucose tolerance tests by other investigators (eg. [4]). The average meal size received during this study was 0.38 g. The small size of this meal facilitated relatively uniform and complete consumption of the meal by all animals. In addition, a small meal size reflects the nibbling nature of a rat's natural eating pattern and is therefore physiologically appropriate for use in a rodent model.

Over the 2 h postprandial period, there was approximately a 30% reduction in the amount of lipogenesis, exclusively in white adipose tissue, in response to a RS diet relative to a DS diet. This number is reflective of data in humans which shows a 20–30% increase in postprandial fat oxidation following a RS meal [3]. As rats are 'nibblers', they will conceivably consume a meal of the size fed in this study many times over the course of a day. There fore, the observed 30% reduction in lipid synthesis in response to a RS diet could reduce adipose tissue mass in the long-term.

Further studies need to be conducted to elucidate the mechanism/s behind the observed decrease in lipogenesis in WAT in response to RS consumption. As no differences in circulating plasma triglyceride or NEFA concentrations was observed, it is possible that differences in circulating insulin concentration play a role in this effect. We have previously shown that RS consumption, at the level fed in the present study, decreases circulating plasma insulin without changing plasma glucose concentration in rats [6]. It could be argued that this attenuation in insulin concentration due to RS consumption is effecting WAT lipogenic enzyme activity as it has previously been shown that these enzymes are more insulin responsive in WAT than liver which could explain the tissue specificity of the observed response.

Kabir et al [2] have previously reported that long-term feeding of a high RS diet reduces fat cell size relative to a high DS diet. Data presented here indicate that a high RS meal reduces total fat synthesis in white adipose tissue following a meal relative to a high DS meal. This decrease in lipid synthesis could indeed contribute to control of fat cell size in RS fed rats in the long-term. Decreased lipogenesis selectively in WAT and a reduction in adipocyte cell size in response to RS feeding have broad implications for the treatment and prevention of obesity. Furthermore, increasing the RS intake of a typical Western diet would be easy to achieve through the consumption of natural foods with a high RS content and/or the consumption of commercially produced foods which are fortified with high RS starches, such as breads, pasta, and cereals. RS can even be easily incorporated into foods such as candy and pizza dough, making increased consumption an achievable possibility.

## Abbreviations

RS, resistant starch

DS, digestible starch

WAT, white adipose tissue

BAT, brown adipose tissue

NEFA, non-esterified fatty acid

## Competing interests

The author(s) declare that they have no competing interests.

## Authors' contributions

JH conceived of the study design and was responsible for overall study coordination, conducting rat meal tests/sacrifice, data analysis, and manuscript preparation. MB was responsible for conducting rat meal tests/sacrifice, statistical analyses, and contributed to manuscript preparation. LS conducted rat tissue extractions and contributed to the study design and manuscript preparation.
